# Rationale and design of the Diet Restriction and Exercise-induced Adaptations in Metastatic breast cancer (DREAM) study: a 2-arm, parallel-group, phase II, randomized control trial of a short-term, calorie-restricted, and ketogenic diet plus exercise during intravenous chemotherapy versus usual care

**DOI:** 10.1186/s12885-021-08808-2

**Published:** 2021-10-10

**Authors:** Amy A. Kirkham, Karen King, Anil A. Joy, André B. Pelletier, John R. Mackey, Kelvin Young, Xiaofu Zhu, Judith Meza-Junco, Sanraj K. Basi, Julie Price Hiller, Tina Brkin, Bonnie Michalowski, Edith Pituskin, D. Ian Paterson, Kerry S. Courneya, Richard B. Thompson, Carla M. Prado

**Affiliations:** 1 Faculty of Kinesiology & Physical Education, University of Toronto, 422, 100 Devonshire Pl, Toronto, ON M5S 2C9 Canada; 2 Cross Cancer Institute, Edmonton, AB Canada; 3 University of Alberta, Edmonton, AB Canada

**Keywords:** Breast cancer, Metastatic, Chemotherapy, Exercise, Ketogenic, Calorie restriction, Nutrition

## Abstract

**Background:**

An underlying cause of solid tumor resistance to chemotherapy treatment is diminished tumor blood supply, which leads to a hypoxic microenvironment, dependence on anaerobic energy metabolism, and impaired delivery of intravenous treatments. Preclinical data suggest that dietary strategies of caloric restriction and low-carbohydrate intake can inhibit glycolysis, while acute exercise can transiently enhance blood flow to the tumor and reduce hypoxia. The Diet Restriction and Exercise-induced Adaptations in Metastatic Breast Cancer (DREAM) study will compare the effects of a short-term, 50% calorie-restricted and ketogenic diet combined with aerobic exercise performed during intravenous chemotherapy treatment to usual care on changes in tumor burden, treatment side effects, and quality of life.

**Methods:**

Fifty patients with measurable metastases and primary breast cancer starting a new line of intravenous chemotherapy will be randomly assigned to usual care or the combined diet and exercise intervention. Participants assigned to the intervention group will be provided with food consisting of 50% of measured calorie needs with 80% of calories from fat and ≤ 10% from carbohydrates for 48–72 h prior to each chemotherapy treatment and will perform 30–60 min of moderate-intensity cycle ergometer exercise during each chemotherapy infusion, for up to six treatment cycles. The diet and exercise durations will be adapted for each chemotherapy protocol. Tumor burden will be assessed by change in target lesion size using axial computed tomography (primary outcome) and magnetic resonance imaging (MRI)-derived apparent diffusion coefficient (secondary outcome) after up to six treatments. Tertiary outcomes will include quantitative MRI markers of treatment toxicity to the heart, thigh skeletal muscle, and liver, and patient-reported symptoms and quality of life. Exploratory outcome measures include progression-free and overall survival.

**Discussion:**

The DREAM study will test a novel, short-term diet and exercise intervention that is targeted to mechanisms of tumor resistance to chemotherapy. A reduction in lesion size is likely to translate to improved cancer outcomes including disease progression and overall survival. Furthermore, a lifestyle intervention may empower patients with metastatic breast cancer by actively engaging them to play a key role in their treatment.

**Trial registration:**

ClinicalTrials.gov, NCT03795493, registered 7 January, 2019.

## Background

Individuals with metastatic breast cancer have a median overall survival of 23–37 months after diagnosis, a 26% 5-year survival rate, and exert a health care cost that is 2.2–2.5-fold higher than those diagnosed with early-stage breast cancer [1–4]. Among patients with metastatic cancer, tumor resistance to chemotherapy accounts for 90% of cases where treatment fails, ultimately leading to death [5]. In solid tumors, vascular impairment that results in poor blood supply is an important mechanism of resistance to chemotherapy and also promotes metastasis [6]. Poorly perfused tumors experience diminished delivery of oxygen and blood-borne (i.e., systemic) therapies including chemotherapy. Tumors lacking adequate oxygen delivery adapt to the resultant hypoxic environment by metabolizing glucose without oxygen (anaerobically) to produce energy. In addition to the rapidly dividing cells characteristic of malignancy, solid tumors also contain regions of slowly proliferating malignant cells that have a hypoxic microenvironment [7]. The inability of chemotherapy to target slowly proliferating cell regions, the degree of tumor hypoxia, and the reduced uptake of therapeutic agents, are all associated with tumor resistance to chemotherapy [7].

Important potential therapeutic goals to combat these mechanisms of tumor resistance include increasing tumor blood flow to reduce hypoxia and increase delivery of treatment, as well as inhibition of anaerobic glucose metabolism (i.e., glycolysis) to target the slowly proliferating tumor cells. Indeed, drug-induced improvement in arterial blood flow to the tumor combined with chemoradiation has been shown to correlate with improved overall survival [8, 9]. Accordingly, the combination of chemotherapy treatment with inhibition of glycolysis would be expected to improve overall therapeutic efficacy [7].

Lifestyle interventions, including diet and exercise when combined with chemotherapy can target these mechanisms as a potential strategy to enhance treatment efficacy. For example, acute exercise can exploit the impaired vascular function of tumors to enhance blood flow to the tumor by over 200% compared to a resting state in rodents [10, 11]. In tissues with normal vascular function, acute exercise induces a tissue-specific redistribution of blood flow via dilatation of blood vessels to increase flow to active tissues (i.e., active skeletal muscles), and constriction of vessels to reduce flow to inactive tissues (i.e., viscera, brain etc.) Acute exercise results in increased blood to tumors because their vessels do not have the ability to constrict, even without alteration in blood flow to the tumor's host tissue [10]. These data indicate that acute exercise can increase blood flow to tumors located in tissues where exercise does not normally cause increased flow such as the breast or internal organs. As a result of increased blood flow, oxygen delivery is also increased to the tumor, and acute exercise also reduces hypoxia in the tumor microenvironment by 50% [10, 11]. These findings suggest that acute exercise used in combination with intravenous chemotherapy would enhance chemotherapy delivery to the tumor and reduce tumor hypoxia [12]. The safety and feasibility of acute exercise during chemotherapy infusions in humans has been demonstrated in two small pilot studies [11, 12].

Both caloric restriction (i.e., a reduction in daily calorie intake without malnutrition) and a high-fat / low-carbohydrate or ketogenic diet can inhibit glycolysis [13, 14]. As such, combining both nutritional approaches could be a feasible and effective way to target this aspect of tumor resistance. Preclinical data suggest that short periods of fasting or caloric restriction combined with chemotherapy treatment inhibit tumor growth, enhance chemotherapy efficacy, and reduce side effects [15–19]. Furthermore, nutrient deprivation increases the resistance of healthy cells (but not cancer cells) to the damaging effects of oxidative stress by 1000-fold [15, 20]. This phenomenon, called ‘selective stress resistance’ could reduce treatment toxicity and maximize the relative dose intensity. Preliminary data suggest that short-term fasting or caloric restriction is safe, feasible, and shows promise for reducing treatment toxicity in patients [21]. Combining caloric restriction with aerobic exercise has been shown to down-regulate more cancer signalling pathways than either intervention alone in rodents [22]. Additionally, this combination has been shown to have synergistic effects in non-cancer clinical populations on outcomes relevant to cancer including body composition [23], aerobic fitness [24], fasting insulin and glucose [25], and IGF-1 [26].

The purpose of the Diet Restriction and Exercise-induced Adaptations in Metastatic Breast Cancer (DREAM) study is to compare the effects of a 50% calorie-restricted and ketogenic diet for 48–72 h prior to each chemotherapy treatment combined with aerobic exercise performed during intravenous chemotherapy on changes in tumor burden (primary aim) compared to usual care. The study will also evaluate the effects on quantitative treatment side effects to heart, skeletal muscle, and liver, as well as patient-reported quality of life and symptoms. We hypothesize that the intervention will enhance tumor response to chemotherapy without increasing side effects. Exploratory aims include assessing the impact of the intervention on progression-free and overall survival.

## Methods

### Design and ethics

**Fig. 1:**
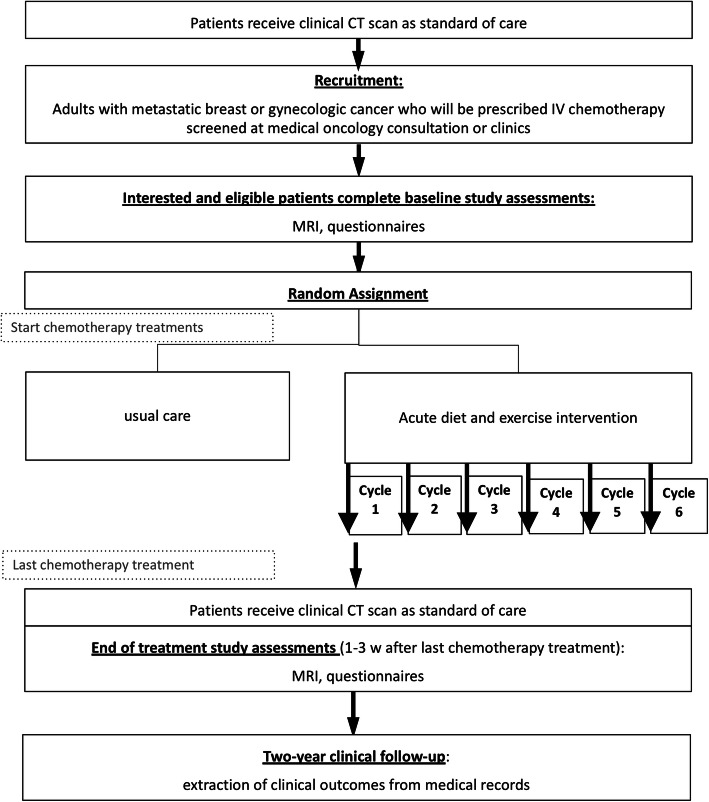
Study design. CT = computed tomography; w = weeks

The DREAM study is a two-arm, parallel-group, phase II randomized controlled trial (ClinicalTrials.gov Identifier: NCT03795493). A summary of the study design is illustrated in Fig. 1. The Health Research Ethics Board of Alberta Cancer Committee approved this study (HREBA.CC-18-0657), and all participants will provide written informed consent. The study is taking place at a single study site in Edmonton, Canada at the University of Alberta and the Cross Cancer Institute, a tertiary care cancer treatment hospital. The study team will communicate any important protocol modifications to the trial registry and research ethics board.

### Randomization

The randomization will employ a permutated block design with random block sizes of four and six, and a 1:1 allocation ratio to usual care or the combined diet and exercise intervention. Randomization will be stratified by the active line of intravenous chemotherapy (i.e., first, second, or ≥ third), as further lines of treatment are known to confer shorter periods of disease control compared to earlier lines [27]. An external party to the study will use a random spreadsheet function to generate the randomization sequence and will place the group assignment into sequentially numbered, sealed, opaque envelopes. The study coordinator will gain written consent from the participants and perform the randomization following completion of all baseline assessments.

### Blinding

It will not be possible to blind participants to their group assignment. Group assignment preference could influence patient-reported outcomes and adherence but is unlikely to influence physical outcome measures. All study outcome assessment and analyses with subjective input will be performed blinded to group assignment.

### Patients and recruitment

Participant eligibility criteria are listed in Table 1. The study coordinator will screen the breast cancer medical oncology clinics at the cancer treatment center using electronic medical records and notify the treating medical oncologist of potential eligible participants. If the treating medical oncologist approves of the patient’s participation, they will obtain consent from the patient for the study coordinator to contact them. The treating medical oncologists can also alert the study team to potential participants in their care as they become eligible (i.e., switching to a new treatment line).

**Table 1:** Participant eligibility criteria

Inclusion criteria	Exclusion criteria
• Age > 18 years • Able to communicate and read in English • Diagnosis of stage IV (metastatic) breast cancer • Starting (or having only received one treatment of) a new line of any type of intravenously administered chemotherapy • Treating medical oncologist approval • Measurable metastases^a^ • Willing and able to adhere to the study interventions and assessments • ECOG < 3	• Limitations to sustained exercise • Clinical evidence of cachexia (oncologist’s discretion) • Body mass > 109 kg (weight limit on cycle ergometer) • Supplemental oxygen requirement • Uncontrolled pleural effusions (oncologist’s discretion) • Severe food allergies or diet restrictions • History of eating disorder (diagnosed or self-reported) • Unable to provide informed consent • Type 1 or 2 diabetes mellitus • Bilirubin > 30 umol/L • Creatinine > 120 umol/L • Contraindications to 3 T MRI for research purposes (e.g., pregnancy, pacemaker, magnetic implant)

^a^Measurable is defined according to RECIST guidelines as computed tomography evidence of at least one of: 1) tumor lesions with a long-axis diameter of ≥10 mm; or 2) malignant lymph nodes with a short-axis ≥ 15 mm

### Interventions

#### Both groups

As a strategy to manage the expected self- or oncologist-selection bias for a study involving diet and exercise, and to enhance recruitment and retention, all participants, regardless of group assignment, will receive a one-time phone consultation with a registered dietitian and with a certified exercise physiologist. The phone consultations will be scheduled within the first chemotherapy cycle after randomization. These professionals will provide general advice in line with the Canadian Cancer Society guidelines on nutrition and exercise. The dietitian will utilize the resting energy expenditure (REE) data collected on each participant (described below) to individualize counselling. The exercise physiologist will ask the participant about physical activity habits and experience and develop individualized recommendations consistent with the guidelines.

#### Diet and exercise intervention group

Participants assigned to the intervention group will consume a provided diet for 48–72 h prior to each chemotherapy treatment and perform cycle ergometer exercise during each chemotherapy infusion, up to a maximum of six treatment cycles of the same line, in addition to usual cancer care. If the treatment line changes at the discretion of the treating medical oncologist prior to the completion of the six cycles, the intervention will be stopped, and the participant will complete the end of study assessments.

##### Diet intervention

In the 72 h prior to each scheduled start time of chemotherapy treatment, participants will be asked to consume only the provided study diet. When there are < 7 days between infusions (i.e., weekly protocols), participants will follow the diet protocol for 48 h prior to that treatment to reduce the number of days in consecutive weeks spent in a calorie deficit to avoid significant weight loss. Participants will choose three meals (breakfast, lunch, dinner) and two snacks per planned day of the diet from a study menu with 3–4 options for each meal for each treatment (meal descriptions provided in Table 2). The study team designed the menu such that the total caloric content will provide 50% of daily energy expenditure (EE). Daily EE is measured in intervention participants prior to the first treatment cycle on intervention as 1.4 multiplied by the REE (assessment described below). The three meals are isocaloric, with each representing 25% of daily EE and each snack representing 12.5%. Additionally, each meal and snack will have a macronutrient composition designed to induce ketosis, with 80% of the calories coming from fat, and 10% coming from each protein and carbohydrates calculated using Food Processor SQL v11.0.3 (ESHA Research, Salem, OR) [28]. When combined with 50% caloric restriction, the 10% carbohydrate composition will provide 22–30 g/day of carbohydrates in the range of daily EE for this population (50% daily EE expected to range from 900 to 1200 kcal based on past experience). The study team will freshly prepare the food in a metabolic kitchen. Each meal and snack will be individually packaged, labelled, and placed in an insulated cooler for pickup by the participant. Participants will also be asked to complete a study diary describing any dietary deviation from the provided food and any adverse nutrition impact symptoms experienced (e.g., light-headedness, fatigue, nausea, diarrhea, dizziness). As a further measure of protocol fidelity, participants will be given a validated handheld monitor (Precision Xtra, MN 98814, Abbott, Alameda, USA) [29] to measure their blood glucose and blood ketones after following the diet for 24, 48, and 72 h (sample performed after 2 h of water-only fast).

**Table 2:** Study meal and snack options designed to each contain macronutrient ratios of approximately 80% fat, 10% protein, and 10% carbohydrate

Description	Ingredients	Dietary Preferences
**Breakfast Options**		
Omelet	Egg yolk, bacon, spinach, olive oil, mandarin orange (on the side)	GF, DF
Hash browns and tuna pâté	Canned tuna, cream cheese, chives, white potato, butter	GF
Assorted	Peanut butter, avocado, bacon	GF, DF
**Lunch Options**		
Salad	Lettuce, bacon, feta, tomato, croutons, hard-boiled egg, ranch dressing, olive oil	
Vegetable soup	Celery, broccoli, carrots, cauliflower, onion, cheddar cheese, whipping cream, olive oil	VG, GF
Zucchini Bolognese	Zucchini spirals, ground beef, tomato sauce, onion, olive oil, parmesan cheese	GF
**Dinner Options**		
Chicken casserole	Chicken thigh, cauliflower, broccoli, butter, lemon juice, chicken broth, whipping cream, breadcrumbs	
Pumpkin and sausage soup	Pureed pumpkin, pork sausage, onion, whipping cream, chicken broth, curry powder, olive oil	GF
Zucchini alfredo soup	Zucchini, butter, parmesan cheese, flour, whipping cream	VG
Cauliflower pizza crust with guacamole	Avocado, olive oil, lime juice, cauliflower, egg, cheddar cheese	VG, GF
**Snack Options**		
Yogurt	Vanilla yogurt, almonds, coconut oil	VG, GF
Veggies and dip	Chopped carrots and celery, ranch dressing dip, cheese string	VG, GF
Nuts & peanut butter	Walnuts, peanut butter	VG, GF, DF
Salad sandwich	Romain lettuce, avocado, feta, tomatoes, olive oil	VG, GF

Notes: *GF* Gluten-free, *DF* Dairy-free, *VG* vegetarian

##### Exercise intervention

During the chemotherapy infusion, participants will perform aerobic exercise on a recumbent cycle ergometer (Elite Total Body Recumbent Bike 15–9100, Stamina Products Inc., Springfield, MO) in a designated research area of the chemotherapy day unit with the standard medical supervision for chemotherapy plus exercise supervision by a certified exercise physiologist. The chemotherapy nurse assigned to the participant for that day will perform the pre-treatment assessment and establish the intravenous line access (either via insertion of peripheral venous cannula or use of established tunneled central venous catheter) in the chemotherapy chair before the participant is carefully transferred to the cycle ergometer for exercise. The pre-exercise preparation for common chemotherapy protocols is described in Table 3. For example, for taxane-based chemotherapy protocols with a greater risk of an acute adverse reaction, the participant will remain seated in the chair for the first 15 min of the infusion and then be carefully moved to the cycle ergometer. During the exercise session, the exercise physiologist will monitor blood pressure (automated cuff in chemotherapy unit), heart rate (Polar OH1+ armband monitor, Polar Canada, Quebec, Canada) and arterial oxygen saturation (finger clip oximeter), and subjective rating of perceived exertion. Based on the rodent proof-of-concept studies, moderate-intensity exercise is sufficient to enhance tumor blood flow substantially [10, 11]. Moderate-intensity will be prescribed for the participants as 40 to 60% of age-predicted heart rate reserve (calculated as: [220 - age - resting heart rate (measured in chemotherapy chair)] * intensity + resting heart rate). Participants will be encouraged to work toward the upper end of the intensity range, dependent on abilities. The session duration will be adapted to the chemotherapy protocol to ensure the generalizability of the intervention (Table 3). Typically, sessions will consist of a 5-min warm-up, 30–60 min at the target heart rate and a 5-min cool-down. An additional 5–10 min of warm-up or cool-down may be performed on case-by-case basis due to delays to infusion initiation or completion (e.g., unit patient load, post-treatment intravenous flush length). If required, 2-min breaks will be provided up to a maximum of every 8 min to enable rest but limit the time with reduced blood flow.

**Table 3:** Example exercise protocols for different chemotherapy regimens

Chemotherapy Protocol	Intravenous Delivery Length and Method	Pre-Exercise Preparation	Exercise Duration
Single-agent docetaxel (1 per 21-day cycle)	60-min infusion	• IV access established in chair • 15-min rest after infusion start • Patient carefully transferred to cycle ergometer • Warm-up initiated	• 5-min warm-up • 35 min at target heart rate • 5–10-min cool-down
Pertuzumab + trastuzumab + docetaxel (1 per 21-day cycle)	Pertuzumab + trastuzumab infusion over ~ 5 h for loading dose/ ~ 3 h for the second dose/ 1.5–2.5 h for subsequent doses, followed by docetaxel 60-min infusion	• Pertuzumab + trastuzumab infused at rest in chair • Docetaxel infusion initiated • 15-min rest • Patient carefully transferred to cycle ergometer • Warm-up initiated	• 5-min warm-up • 35 min at target heart rate • 5–10-min cool-down
Single-agent paclitaxel (3 per 28-day cycle)	60-min infusion	• IV access established in chair • Infusion initiated • 15-min rest • Patient carefully transferred to cycle ergometer • Warm-up initiated	• 5-min warm-up • 35 min at target heart rate • 5–10-min cool-down
Single-agent nanoparticle albumin–bound (nab) paclitaxel (1 per 21-day or 3 per 28-day cycle)	30-min infusion	• IV access established in chair • Infusion initiated • Patient carefully transferred to cycle ergometer • Warm-up & infusion initiated	• 5-min warm-up • 25 min at target heart rate • 5–10-min cool-down including IV bag flush
Atezolizumab + nab-paclitaxel (2 per 28-day cycle + 1 nab-paclitaxel only)	Atezolizumab infusion over 60 min for loading dose/ 30 min for subsequent doses, followed by nab-paclitaxel 30-min infusion	• IV access established in chair • Atezolizumab infused in chair • Patient carefully transferred to cycle ergometer • Warm-up & infusion initiated	• 5-min warm-up • 25 min at target heart rate • 5–10-min cool-down including IV bag flush
Atezolizumab + paclitaxel (2 per 28-day cycle + 1 paclitaxel only)	Atezolizumab infusion over 60 min for loading dose/ 30 min for subsequent doses, followed by paclitaxel 60-min infusion	• IV access established in chair • Atezolizumab infused in chair • Paclitaxel infusion initiated • 15-min rest • Patient carefully transferred to cycle ergometer • Warm-up initiated	• 5-min warm-up • 35 min at target heart rate • 5–10-min cool-down
Trastuzumab-emtansine (1 per 21-day cycle)	30-min infusion	• IV access established in chair • Infusion initiated • Patient carefully transferred to cycle ergometer • Warm-up initiated	• 5-min warm-up • 25 min at target heart rate • 5–10-min cool-down
Single-agent eribulin (2 per 21-day cycle)	2–5-min push injection	• IV access established in chair • Saline infusion • Patient carefully transferred to cycle ergometer • Warm-up completed • Push injection initiated	• 5–10-min warm-up prior to push injection • Target heart rate maintained during push injection • 10–20-min at target heart rate post-push injection • 5-min cool-down
Single-agent vinorelbine (2 per 21-day cycle)	10-min infusion	• IV access established in chair • Saline infusion • Patient carefully transferred to cycle ergometer • Warm-up completed • Infusion started	• 5–10-min warm-up prior to infusion • Target heart rate maintained during infusion • 10–20-min at target heart rate post-infusion • 5-min cool-down
Single-agent carboplatin (1 per 21-day cycle)	45-min infusion	• IV access established in chair • Infusion initiated • Patient carefully transferred to cycle ergometer • Warm-up initiated	• 5-min warm-up • 35 min at target heart rate • 5-min cool-down
Gemcitabine + cisplatin (2 per 21-day cycle)	Gemcitabine infusion over 30–60 min, followed by saline infusion over 30–60 min, followed by 60-min cisplatin infusion	• IV access established in chair • Gemcitabine and saline infused in chair • Patient carefully transferred to cycle ergometer • Warm-up & cisplatin infusion initiated	• 5-min warm-up • 50 min at target heart rate • 5-min cool-down
Single-agent doxorubicin (1 or 3 per 21-day cycle)	4–10-min push injection	• IV access established in chair • Saline infusion • Patient carefully transferred to cycle ergometer • Warm-up completed • Push injection started	• 5–10-min warm-up prior to push injection • Target heart rate maintained during push injection • 10–20 min at target heart rate post-push injection • 5-min cool-down
Doxorubicin + fluorouracil + cyclophosphamide (1 per 21-day cycle)	Doxorubicin 4–10-min push injection, followed by fluorouracil 2–5-min push injection, followed by cyclophosphamide 30-min infusion	• IV access established in chair • Saline infusion • Patient carefully transferred to cycle ergometer • Warm-up & first push injection initiated	• 5-min warm-up • 40 min at target heart rate • 5-min cool-down

*Abbreviations*: *IV* Intravenous. Notes: With intravenous infusion delivery method, the chemotherapy is slowly injected from a plastic bag through tubing into the vein with the flow rate controlled by an infusion pump. With intravenous push injection deliver method, the chemotherapy is delivered quickly from a syringe directly into the vein

#### Usual care group

Participants randomized to the usual care group will receive usual cancer care. Additionally, to maintain study engagement and control for social interaction, the study coordinator will meet participants at the cancer treatment center prior to each chemotherapy cycle. The study coordinator will collect the Rotterdam treatment symptoms checklist and blood glucose and ketone levels during these visits to assess symptoms and contamination (i.e., participants following the ketogenic diet on their own).

### Outcome measures

The study outcome measures and their timing are summarized in Table 4. In line with an intention-to-treat approach, all outcome measures will be completed for participants who discontinue or deviate from the intervention protocols, where possible. The primary outcome for determining the efficacy of the intervention on tumor response to chemotherapy is change in target lesion size measured by clinical computerized tomography (CT) scan after up to six chemotherapy treatments. The secondary tumor response outcomes will be change in MRI-derived water apparent diffusion coefficient (ADC) within the target lesion after up to six treatments, a novel marker of treatment-induced tumor regression [30, 31], and change in target lesion size measured by clinical CT after three treatments. It is well established that the degree of tumor size change during chemotherapy is a strong independent predictor of overall, disease-free and recurrence-free survival [32–35]. The Response Evaluation Criteria in Solid Tumors (RECIST) Working Group has defined objective criteria to categorize tumor response to therapy for clinical trials as partial, complete, progressive, or stable [36]. However, tumor size will be used as a continuous variable rather than the RECIST categorical variable to enhance statistical power to detect an effect of the intervention with a phase II sample size.

**Table 4:** Summary of study outcome measures and timing of assessment

	Assessment Method	Pre cycle 1	Post cycle 3	Post cycle 6	2-year follow-up
**Primary Outcome**					
Target lesion size change after 6 cycles (mm)	CT Scan	X		X	
**Secondary Outcomes**					
Target lesion apparent diffusion coefficient (mm^2^/s)	MRI	X		X	
Target lesion size change after 3 cycles (mm)	CT Scan	X	X		
**Tertiary Outcomes**					
Left ventricular ejection fraction (%)	MRI	X		X	
Left ventricular global longitudinal strain (%)	MRI	X		X	
Left ventricular mass (g/m^2^)	MRI	X		X	
Liver fat fraction (%)	MRI	X		X	
Liver T_1_ time (ms)	MRI	X		X	
Thigh skeletal muscle T_1_ time (ms)	MRI	X		X	
Thigh skeletal muscle volume (mL)	MRI	X		X	
Thigh skeletal muscle fat fraction (%)	MRI	X		X	
Treatment symptoms^a^	Rotterdam Symptom Checklist	X	X	X	
Quality of life	FACT-Fatigue Questionnaire	X	X	X	
Fatigue	FACT-Fatigue Questionnaire	X	X	X	
**Exploratory Outcomes**					
Progression-free survival (months)	Medical records				X
Overall survival (months)	Medical records				X

^a^ Treatment symptoms are assessed after every cycle

Tertiary outcomes will include quantitative and patient-reported treatment side effects. MRI will be used to assess quantitative side effects including markers of cardiotoxicity (left ventricular ejection fraction, longitudinal strain, and mass), and markers of thigh skeletal muscle edema, volume, and quality, and markers of liver steatosis, edema, and fibrosis. These tissues are targeted based on their established roles in the uptake or metabolism of chemotherapy, performance of cycle ergometer exercise, or both. Validated questionnaires will be used to assess patient-reported treatment symptoms and quality of life. Tertiary outcomes will be compared between groups at each time point of assessment if baseline values are balanced between groups; otherwise change scores will be used. Exploratory outcome measures will include progression-free survival and overall survival at 2 years.

### Outcome measure collection methods

The cancer treatment center will perform CT scans as standard of care prior to the first line of a new treatment, after three treatment cycles, and after completion of up to six treatment cycles or prior to changing lines of therapy. Patients are administered an oral contrast agent prior to the scan and receive an intravenous iodinated contrast agent during the examination. Scans are performed on a 64-slice CT machine (SOMATOM Definition Flash, Siemens, Forchheim, Germany), in a cranio-caudal direction starting from the lung apices to below the symphysis pubis in 5 mm slice thickness. A single observer on the study team who is blinded to group assignment will analyze all CT images to control for potential differences in measurement between radiologists. Analysis of the target lesion size will be performed with a custom Matlab program (The Mathworks, Natick, USA). The study team will use the RECIST guidelines to select the target lesions at baseline by the following criteria: 1) up to five total lesions with a limit of two per organ; 2) each included lesion must have a long-axis diameter of ≥10 mm and included malignant lymph nodes must have a short-axis ≥ 15 mm.

**Fig. 2:**
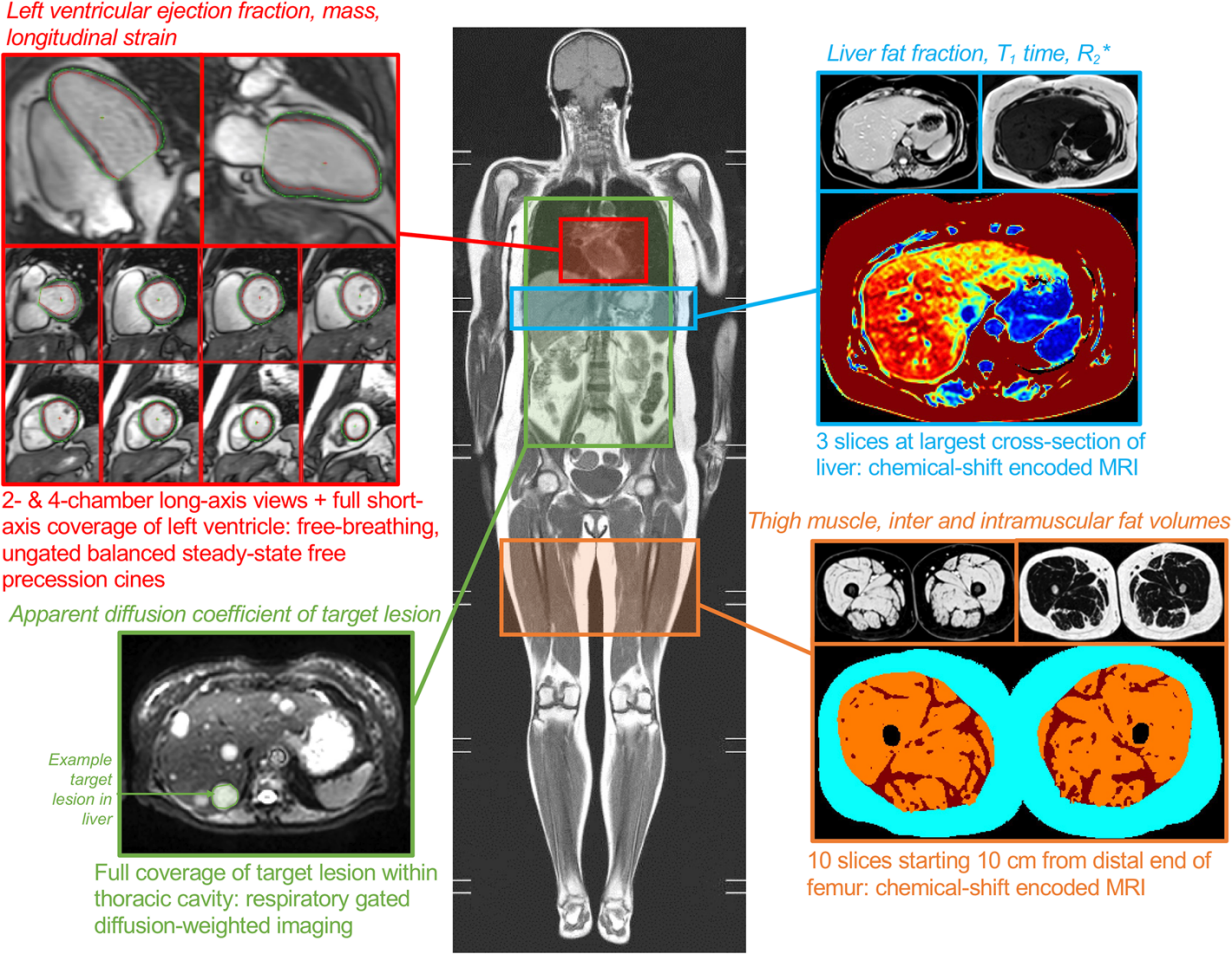
Magnetic resonance imaging protocol

Prior to randomization and again after up to six chemotherapy cycles, we will perform a research-based, 30-min, non-contrast, MRI examination to assess lesion ADC and quantitative metrics of the heart, skeletal muscle, and liver (Fig. 2). The scans will be performed on a 3 T Siemens Prisma scanner (Siemens, Erlangen, Germany) with 36-element chest/back array for signal reception. The target lesion ADC will be acquired by a respiratory navigator-gated diffusion-weighted imaging sequence (typical parameters: field of view (FOV) = 380 × 306 mm, matrix = 134 × 108, flip angle = 90 °, 35 slices, 5 mm slice thickness with 1 mm gap, TE = 37.0 ms, TR = 2 s, 2488 Hz/pixel receiver bandwidth, GRAPPA = 3, *b* value of 0, 50, 150 and 500 s• mm^− 2^ with 2, 2, 2, and 8 averages, respectively, SPAIR fat suppression). A single observer on the study team who is blinded to group assignment will measure the ADC in the target lesion using a custom Matlab program (The Mathworks, Natick, USA).

We will acquire skeletal muscle and liver images with the PROFIT1 chemical-shift encoded MRI (CSE-MRI) method for the simultaneous imaging of proton density fat fraction (fat content), R_2_* (a correlate of iron content), and T_1_ time (associated with water content, edema, and potential fibrosis of the cells), as previously described [37]. For the liver, we will acquire three slices (6 mm thickness, 12 mm gap) centered at the largest cross-section of the liver during an end-expiration breath-hold (typical parameters: FOV = 450 × 280 mm, matrix = 160 × 88, flip angle = 30 °, GRAPPA = 2, TE = 1,14, 2.76, 4,38, 6.00, and 7.62 ms, TR = 30 ms). For the liver image analysis, a single study team observer will use custom software to manually trace the liver on each slice, with automated removal of the blood vessels. We will manually select healthy liver tissue (i.e., not containing metastatic lesion) in each slice to calculate the fat fraction, R_2_*, and T_1_ time. In the skeletal muscle, we will acquire five 4.0 mm interleaved axial orientation slices (12 mm gap) of both thighs starting from 10 cm proximal to the most distal point of the femur (typical parameters: FOV = 420 × 210 mm, matrix = 224 × 112, flip angle = 40 °, GRAPPA = 2, TE = 2.51, 3.51, 4.51, 4.78, 5.78 and 6.78 ms, TR = 55 ms). We will use custom software to automatically segment the subcutaneous fat, muscle, intermuscular fat (IMF) areas and quantify total volumes using the disk summation method for the thigh image analysis. We will calculate the ratio of IMF to skeletal muscle and the skeletal muscle fat fraction (IMF/(IMF + muscle)*100%) as measures of muscle quality. T_1_ time of the thigh muscle will be averaged across the muscle area.

We will acquire cardiac images as real-time, free-breathing balanced steady-state free precession cines of the left ventricle (typical imaging parameters: FOV = 440x260mm, matrix = 224 × 90, flip angle = 30 °, GRAPPA = 3, TE = 1.0 ms, TR = 38 ms, 60 images/slice, 8 mm slice thickness, 2 mm gap). We will measure left ventricular ejection fraction, global longitudinal strain and mass from these images using custom in-house software as previously described [38].

Cancer-specific quality of life and fatigue will be assessed by the Functional Assessment of Cancer Therapy – Fatigue (FACT-F) [39] prior to randomization, after three and six treatment cycles. Treatment symptoms will be assessed by the Rotterdam Symptom Checklist [40], prior to each treatment cycle as a method for study staff to closely track ongoing symptoms. Mortality and progression data will be extracted from patient medical records.

### Descriptive and confounding variables collection

A brief researcher-designed questionnaire will be used to collect demographic information. We will manually extract medical history including diagnosis and relevant treatment-related information such as protocol, dosage, delays, and hospitalizations from patient medical records.

We will measure REE before or shortly after randomization in all participants to accurately prescribe the calorie restriction in the intervention group and to provide information to the registered dietitian for the phone consultation. We will use indirect calorimetry with a metabolic cart and ventilated hood system (VMax Encore, Vyaire Medical, Yorba Linda, CA, USA) in the morning after a 12-h water-only fast. We will perform gas and flow meter calibration prior to each test according to manufacturer instructions. Participants will rest supine for 10 min, then a clear dome and canopy will be placed over their head and shoulders. We will use only steady-state values (defined as ≤10% variation over 5 min) of oxygen consumption and carbon dioxide production to calculate REE. After collecting 15 min of steady-state data or 30 min total, we will terminate the test.

The Recent Physical Activity Questionnaire [41] and the Nutrition Quest Brief Food Questionnaire (Berkley Analytics Inc., www.NutritionQuest.com) will be used to assess self-reported exercise and general nutrition habits for the most recent 3 month period, respectively. Both questionnaires will be administered prior to randomization and again after up to six treatment cycles. To assess intervention acceptability, we will use a modified version of a prospective motivational evaluation of the diet and exercise intervention which asks participants to anticipate how beneficial, enjoyable, supported, motivated, and difficult it would be for them to complete the interventions. After completion of the intervention, the intervention group participants will complete a retrospective motivational evaluation in which they will be asked to report how beneficial, enjoyable, supported, motivated, and difficult it was [42].

### Data management

The confidentiality of enrolled participants will be protected by use of unique, random study identification numbers for each participant used on all study data collection. The study personnel and investigators will securely store de-identified research data on network drives where it will only be accessible by members of the study team and their delegates. All data entered electronically will be double-checked for accuracy.

### Participant reimbursement

Reimbursements will be provided for parking expenses for all study visits, and there is no charge associated with the intervention (including food) and assessments.

### Sample size determination

As no prior studies have measured the combined or independent effects of our interventions on tumor response to chemotherapy, we calculated a sample size intended to capture a clinically relevant effect using G*Power software (version 3.1.9.2, Düsseldorf, Germany) [43]. The RECIST guidelines use a 30% reduction in tumor size from baseline as clinically relevant threshold to indicate a partial response. Based on this, we assumed that a clinically relevant intervention effect size would be a mean of 30 percentage point difference between groups. Taking into account the variation in subtypes of metastatic breast cancer and chemotherapy protocols, we estimated a mean tumor size reduction of − 10 to − 20% for the usual care group, with a wide standard deviation of 35%. Therefore, using a mean − 50% reduction in the intervention group and a standard deviation of 35 would provide an effect size of Cohen’s d = 0.857 (or Cohen’s f = 0.4285). A total sample size of 45 patients with an alpha of 0.05 would provide 80% power to detect this effect size. An additional 10% will be recruited (*n* = 50 total, *n* = 25 per group) to account for withdrawals and mortality during the intervention period.

### Statistical analyses

The primary analyses will be intention-to-treat. Per-protocol analyses will also be performed based on adherence to all aspects of the intervention as well as achievement of ketone production. If substantial differences in adherence to the diet and exercise aspects of the intervention occur, then we will also perform sensitivity analysis to determine the individual impact of each intervention on outcomes. To assess for differences between groups in change in all tumor response outcomes, we will use a one-way analysis of covariance with baseline lesion size as a covariate. Other potential covariates that will be tested for inclusion will be estrogen/ progesterone receptor status, human epidermal growth factor receptor 2 status, age, and number of received treatments during the study period. To assess the effect of the intervention on the questionnaire data, generalized linear mixed models will be used (fixed factors = group, time, group*time; random factor = participant). The Kaplan-Meier estimator and Cox proportional hazard models will be used to compare unadjusted and adjusted survival between groups.

### Data monitoring and adverse events

The low risks associated with the diet and exercise components of the intervention do not necessitate a data monitoring committee. Participants assigned to the intervention group will report non-emergent symptoms or adverse events in their food diary during the diet period. This will be reviewed by the exercise physiologist upon arrival for their exercise session to confirm completion and accuracy. Participants will also have contact info for the study staff during the diet period (including weekends) for any perceived emergent concerns. The exercise physiologist will monitor any symptoms or adverse events which occur during the exercise session. Serious adverse events will be reported to study doctors and the research ethics board.

### Dissemination

The study results will be published in a peer-reviewed clinical journal. Co-authorship will be provided to the study team members who meet the International Committee of Medical Journal Editors criteria for authorship. Participants will receive a lay summary of the study results. The study results will also be disseminated by the study sponsors to their stakeholders. Due to research ethics board requirements, the full dataset will not be publicly available. However, de-identified study data will be provided to interested parties upon reasonable request.

## Discussion

Exercise and diet are attractive supportive therapies for individuals diagnosed with cancer because they are easily accessible, inexpensive, associated with improvements to physical fitness and quality of life, and have few risks or side effects [44]. Nearly all exercise and diet research performed in populations with breast cancer has focused on women with early-stage disease (stages I-III) who have an 89% 5-year survival rate. In contrast, metastatic disease (stage IV) remains incurable, with a 26% 5-year survival rate. The current study proposes a novel, short-term diet and exercise intervention targeting mechanisms of tumor resistance to chemotherapy with the potential to reduce treatment-induced oxidative injury to healthy tissues. The timing of the intervention delivery is optimal for patient adherence as it aligns with the nadir of treatment symptoms (i.e., the end of each chemotherapy cycle) and the acute dose of diet and exercise is more manageable for a wider range of patients than consistent long-term adherence to diet and exercise.

This study design is innovative as it will determine the intervention efficacy in a short-term longitudinal study design rather than reliance on longer-term outcomes that may require several years of follow-up for the primary outcome by utilizing: 1) patients with measurable metastases; and 2) CT for tumor size and MRI for novel measures of tumor response. A reduction in tumor size within the intervention group is expected to translate to improved cancer outcomes including disease progression, overall survival, and quality of life, and thereby will have a direct impact on reducing health care costs. If found to be beneficial, the accessibility, ease of uptake by a wide range of patient demographics, and cost-efficacy of these interventions relative to drug development, increases the feasibility of their widespread adoption within cancer treatment centers as part of supportive care. Furthermore, an acute exercise and caloric restriction intervention may empower patients with metastatic breast cancer by allowing them to be actively engaged with their treatment.

An important practical challenge with the exercise intervention that is also likely to exist at other cancer treatment centers is the scarce space available in the outpatient chemotherapy treatment unit. At our center, there was only one space identified that can accommodate the recumbent cycle ergometer beside the chemotherapy chair without encroaching on the adjacent patient space. Due to the high patient load of the chemotherapy unit, the space is only available to schedule the exercise/treatment sessions on 1 day of the week. Therefore, this limited access precludes the participation of certain patients whose clinic and treatment days cannot practically be modified. A second challenge to the exercise session is adapting the exercise intervention to the various infusion protocols. To enhance generalizability of the study, patients on any intravenous chemotherapy protocol can be included and we adopted the same general principles of exercise prescription across protocols. Finally, an important practical challenge related to diet is the need to include dairy and/or meat products in the ketogenic diet, limiting our ability to include participants on strict vegetarian and vegan diets.

In addition to these practical challenges, limitations of this study include a single site, small sample size, and inability to blind participants and their clinicians to group assignment. The lack of blinding potentially introduces bias in group assignment adherence and our patient-reported outcomes. Furthermore, our primary outcome of tumor size is based on change of size rather than RECIST category of change to enhance statistical power to detect a difference in this early phase II trial. After project completion, the next steps will be to design and perform a phase III clinical trial that is adequately powered to detect a between-group difference in the categorical RECIST guidelines (complete or partial response) as this is the gold standard for assessing outcome of a clinical trial. We will evaluate the feasibility and adherence of the tested interventions in the current study to adapt for further study.

In summary, the DREAM study will evaluate the effect of a short-term diet and exercise approach on chemotherapy treatment response in patients with metastatic breast cancer. These lifestyle interventions are prescribed with the therapeutic goals of inhibition of anaerobic glucose metabolism (by a 50% caloric restricted and ketogenic diet) and increasing tumor blood flow to reduce hypoxia and increase delivery of chemotherapy by aerobic exercise concurrent to intravenous chemotherapy infusion. Targeting mechanisms of tumor resistance to treatment is anticipated to translate to enhanced efficacy of chemotherapy treatment and hence greater tumor response to therapy.

## Data Availability

The datasets used and/or analysed during the current study are available from the corresponding author on reasonable request.

## Abbreviations

ADC: Apparent diffusion coefficient
CT: Computed tomography
DREAM: Diet restriction and exercise adaptations in metastatic breast cancer
EE: Energy expenditure
IMF: Intermuscular fat
IV: Intravenous
MRI: Magnetic resonance imaging
RECIST: Response evaluation criteria in solid tumors
REE: Resting energy expenditure
